# Cross-cultural adaptation and validation of the Conjoint Community Resiliency Assessment Measure (CCRAM) among Chilean adults

**DOI:** 10.3389/fpubh.2026.1866030

**Published:** 2026-06-26

**Authors:** José Sandoval-Díaz, Camila Navarrete-Valladares, Juan Pablo Hidalgo-Ortiz

**Affiliations:** 1 Ñuble Research Center, Universidad del Bio Bío, Chillán, Chile; 2 Department of Legal and Social Sciences, Universidad Adventista de Chile, Chillán, Chile; 3 Department of Engineering and Business, Universidad de las Américas, Concepción, Chile

**Keywords:** Chile, community resilience, confirmatory factor analysis, cross-cultural adaptation, disaster risk reduction, socio-natural hazards, measurement invariance, psychometric validation

## Abstract

**Introduction:**

Community resilience is a relevant construct for public health, disaster risk reduction, and adaptation to socio-environmental crises; however, its measurement remains limited in Latin America.

**Methods:**

This study conducted a cross-cultural adaptation and psychometric validation of the Conjoint Community Resiliency Assessment Measure (CCRAM) among Chilean adults, using two independent subsamples from the Ñuble Region, Chile: one collected in 2023 (*n* = 384) for exploratory analyses and another collected in 2024 (*n* = 545) for confirmatory analyses. Validity evidence based on internal structure was examined through Exploratory Graph Analysis (EGA), Exploratory Factor Analysis (EFA), Confirmatory Factor Analysis (CFA), reliability analysis, discriminant validity assessment, and measurement invariance testing across sex.

**Results:**

The original five-factor structure was not replicated. Instead, the analyses supported an abbreviated 16-item version organized into three correlated factors: Territorial Attachment and Identity, Perceived Local Governance, and Community Social Capital. The final model showed adequate fit, satisfactory reliability, acceptable discriminant validity, and measurement invariance across sex.

**Discussion:**

The findings provide favorable validity evidence and support the use of an abbreviated and contextually adapted version of the CCRAM to assess perceived community resilience among Chilean adults, particularly in relation to disaster risk management and socio-natural hazards.

## 1 Introduction

Community resilience has consolidated itself as a relevant analytical and practical category for the social sciences, public health and disaster risk management (hereinafter, DRM) in recent decades, especially in territories exposed to the climate crisis, socio-environmental conflicts, baseline structural inequalities and territorial vulnerability processes. Community resilience has progressively been incorporated into these contexts, in conceptual frameworks as well as public policy agendas, positioning itself as a strategic axis for disaster risk reduction (hereinafter, DRR), adaptation to climate change and sustainable territorial development (1).

From a community and socio-ecological perspective, the concept of resilience aims to transcend approaches focused on individual adjustment to adversity and become a dynamic, relational, multi-scale, territorial and sociohistorical process that arises from interaction between social actors, institutions, cultural practices and socio-material conditions (2, 3). In this context, community resilience can be defined as the collective capacity to anticipate, adapt and/or transform when faced with critical events, preserving or rebuilding collective well-being, social cohesion and continuity of shared life (3, 4). For the purposes of its measurement within disaster risk reduction, however, this definition requires further specification. First, community resilience does not refer solely to the recovery of collective functioning following a disruptive event, but also to the speed, quality and scope of that recovery (5). Second, it should not be understood as a homogeneous capacity applicable to all types of crises, but rather as a configuration that varies according to the nature of the hazard and the conditions under which communities anticipate, respond to and recover from adversity (2). Thus, a community may demonstrate resilience to socio-natural hazards or disaster-related disruptions without necessarily exhibiting the same level of resilience in response to epidemiological, economic or political crises. In some cases, recovery may also involve strengthening capacities and transforming territorial or institutional conditions to reduce future vulnerabilities, as emphasized in the disaster recovery literature through the concept of *build back better* (6, 7).

Conceptually speaking, community resilience is not a homogenous meta capacity, nor one abstracted from the sociohistorical context (2). Instead, its configuration and deployment depend on situated conditions, including communities’ organizational trajectory, social memory of disasters, the legitimacy of leadership, levels of interpersonal and institutional trust and the relations forged with the state and other DRM-related stakeholders, which have a decisive impact on agency and collective action processes (8). Critical Latin American perspectives have also warned that resilience can become a depoliticizing category when it is separated from the structural processes of vulnerabilization and the state and institutional responsibilities entailed in risk governance. Under such conditions, the emphasis on community adaptation tends to shift responsibility to communities, rendering the sociohistorical inequalities, differential exposure and institutional failures that shape risks invisible (9, 10).

In DRM, community resilience has been progressively incorporated as a strategic component in the prevention, preparation, response and recovery phases. This approach acknowledges communities not as passive recipients of assistance but as central actors in DRR and post-disaster recovery processes (11). Empirical evidence shows that communities with higher levels of resilience tend to have better organizational, communication and coordination capacities, in addition to adaptive recovery trajectories and lower medium- and long-term psychosocial impacts (12).

However, despite the conceptual and empirical expansion of community resilience, its measurement continues to pose a theoretical and methodological challenge. Many of the available instruments have been developed in the Global North, privileging structural or aggregate indicators that do not necessarily manage to capture relevant contextual and psychosocial dimensions, especially in the Global South and Latin America (13). In addition, various instruments operationalize it as a state or result, leaving out its procedural, relational and dynamic nature. This restricts both its conceptual and practical validity, in addition to its usefulness for intervention and the design of contextualized community strengthening strategies (12, 14).

From a contemporary psychometric perspective, measuring community resilience entails balancing the standardization of scores – necessary for their comparison and inferential use – with recognition of the territorial, historical and sociocultural diversity that shapes the construct. According to the Standards for Educational and Psychological Testing (15), validity is not an intrinsic property of the instrument. Instead, the degree to which empirical evidence and the theoretical framework uphold the interpretation of scores for specific uses is. Consequently, cross-cultural adaptation and validation processes gain particular relevance, as they allow examining the construct’s conceptual equivalence, the contextual relevance of the indicators and the soundness of the internal structure in the target population. This entails compiling evidence of validity based on content, the internal structure, the relationship with other variables and score reliability estimates. However, from a situated epistemological perspective, an instrument’s validation does not end with the estimation of factor parameters or reliability coefficients, but rather requires examining the coherence between the construct’s empirical operationalization and its theoretical definition, in addition to its capacity to represent dynamic community processes in specific contexts and/or crises (14).

The Conjoint Community Resiliency Assessment Measure (CCRAM) has been widely used in the context of the growing need to operationalize community resilience. Originally developed in Israel in the framework of research aimed at profiling and predicting perceived community resilience in emergencies, CCRAM was conceived as a multidimensional self-reporting measure (9, 15). Its most widely used formulation, CCRAM-28, considers five dimensions: (i) *leadership*, (ii) *collective effectiveness*, (iii) *preparedness*, (iv) *attachment to place and* (v) *social trust* (9). It was developed in a sequential process combining theoretical foundations, item construction and factor analysis in large community samples. Initially, a bank of 31 items was used, whose empirical assessment allowed identifying six preliminary dimensions (15). Subsequently, Leykin et al. (9) fine-tuned the instrument with exploratory and confirmatory factor analyses of a sample of close to 1,000 participants, giving rise to the version CCRAM-28. Based on this process, a main factor structure of five dimensions composed of 21 items was identified, while the seven remaining reactive items were maintained as complementary contextual indicators. Taking this base, an abbreviated 10-item version (CCRAM-10), aimed at brief assessments and population studies, was also developed with adequate validity indicators and internal consistency (9, 15).

CCRAM has been translated into and examined in different sociocultural contexts over the last decade, including island communities, urban and rural communities in St. Kitts and Nevis (16), China (17), South Korea (18) and the United States (19). This evidence suggests that the instrument has the analytical validity to compare the levels of community resilience in different contexts and examine variations in its dimensions based on exposure to critical events, community experience with risk and participation in local response or intervention bodies. Furthermore, previous studies indicate that some contextual variables – such as residential duration, prior experience with risk situations and participation in community response teams – tend to show more consistent associations with community resilience than certain conventional sociodemographic variables such as gender or occupation (9, 16).

However, the widespread use of CCRAM does not of itself ensure the validity of score interpretations in new sociocultural contexts. Its psychometric transferability requires assessing the conceptual equivalence of the construct, the indicators’ contextual relevance, the stability of the internal structure and measurement of invariance between groups and cultural contexts (13, 20). This need is heightened if one considers that a significant portion of the available psychometric evidence has been produced with WEIRD (Western, Educated, Industrialized, Rich and Democratic) samples, whose sociocultural specificity limits the extrapolation of findings to non-western or socially more heterogeneous populations (21). Thus, despite its international expansion, there is a persistent gap in contextualized psychometric assessment using CCRAM in Latin America, especially in territories characterized by socioeconomic inequality, rurality, institutional centralization of DRM and recurrent exposure to natural risks, such as Chile.

In this context, this study aims to adapt and compile evidence of CCRAM’s validity in the Chilean population. In particular, it proposes examining validity evidence based on internal exploratory and confirmatory structure analyses of independent subsamples, estimating score reliability and assessing factor invariance by sex. Considering the instrument’s development trajectory, an expanded bank of items from its different formulations was used with the purpose of reexamining its internal organization. Thus, the study seeks to provide evidence to support the interpretation of CCRAM scores in the Chilean socioterritorial context and to contribute to the availability of culturally pertinent measures for research and the design of DRM interventions and policies.

## 2 Materials and methods

### 2.1 Design

This study adopted an instrumental design aimed at adapting and assessing CCRAM’s psychometric properties in the Chilean population (22, 23). To this end, two independent samples were used for the exploratory and confirmatory phases of the analysis, respectively. This methodological decision allowed the capitalization of chance that can occur when the derivation and confirmation of the factor solution are carried out on the same sample to be reduced (24).

### 2.2 Procedure

Data production was carried out through in-person implementation of CCRAM in Chile’s Ñuble Region during November 2023 and December 2024. Production was structured in two independent surveys: one in 2023 (*n* = 384) intended for the exploratory phase and another in 2024 (*n* = 545) intended for the confirmatory phase. Previously trained university students were tasked with administering the instrument and the digitalization of data.

### 2.3 Participants

The total sample included 929 adults living in Chile. Of them, 65% were women (*n* = 603), 35% were men (*n* = 325) and one person did not identify with either of these categories. The average age was 40.4 years (*SD* = 17 92; *range* = 18–92).

Two independent subsamples were defined for the psychometric analyses: 2023 (*n* = 384), used for EGA and EFA, and 2024 (*n* = 545), intended for CFA and factor invariance by sex. There were no significant differences between the two groups in terms of age and sex. The sample size was suitable for the factor analyses (24). Preliminary filtering did not reveal invariant response patterns or significant data losses, meaning that no cases were excluded.

### 2.4 Instrument

The Conjoint Community Resiliency Assessment Measure (CCRAM) was used, a self-reporting instrument designed to assess perceived community resilience (9). This study used the Spanish version available at the Ben-Gurion University’s PREPARED Research Center (25), which corresponds to the instrument’s expanded version. This version is composed of 28 items in a five-point Likert response format ranging from 1 (*strongly disagree*) to 5 (*strongly agree*). Its expanded formulation considers a main factor of 21 items distributed across five latent dimensions: (i) *Leadership* (6 items), referring to trust in decision-makers and the performance of local authorities; (ii) *Collective effectiveness* (5 items), associated with mutual support, community involvement and joint capacity for action; (iii) *Preparedness* (4 items), relating to family and community knowledge and preparedness for emergencies; (iv) *Attachment to place* (4 items), associated with the affective bond, the sense of belonging and community pride; and (v) *social trust* (2 items), related to interpersonal trust and the quality of relations among members (9, 25). The seven remaining items are maintained as complementary contextual indicators and are not part of the main factor structure. In the original study, the total scale showed high internal consistency (*α* = 0.92), while the abbreviated version CCRAM-10 displayed adequate internal consistency (*α* = 0.85) (9).

The version used in this study was subjected to linguistic and conceptual adjustments aimed at improving its semantic and contextual relevance to the Chilean population (26). These adaptations included lexical and syntactic simplifications, the adjustment of territorial and institutional references and reformulation of some reactive dimensions to optimize their clarity and adaptation to the application context. Prior to its definitive implementation, the adjusted version was pilot tested on 30 university students to review the items’ understandability and preliminary functioning (23, 27). The adjustments made are detailed in Supplementary Table S1 in the annexes. In addition, three reactive items from the initial formulation were included, thus constituting a bank of 31 items for the internal structure analysis (15). Higher scores indicate greater perceived community resilience.

Lastly, the items are presented in Spanish in the manuscript because that is the language it was applied in. When English translations are included, it is solely for reporting purposes.

### 2.5 Statistical analysis

Statistical analysis was carried out in sequential stages, consistent with contemporary psychometric assessment standards (13), with the purpose of examining the instrument’s internal structure, score reliability and factor invariance. The analytic sequence was designed to progressively examine item performance, estimate the latent dimensionality of the scale, contrast the exploratory solution in an independent sample, and evaluate the reliability, discriminant validity and measurement invariance of the final model.

First, descriptive analyses of the items were performed considering median (*Mdn*), interquartile range (ICR), asymmetry and kurtosis, responding to the ordinal nature of the responses. Furthermore, corrected item-total correlations and multiple squared correlations (R^2^) were calculated as preliminary indicators of discrimination. The bank of items’ internal consistency was estimated using ordinal alpha (*α*), appropriate for Likert scales with ordinal structures (28). Items with corrected item-total correlations below.30 were considered candidates for exclusion.

Second, an exploratory graph analysis (EGA) was applied to estimate the instrument’s latent dimensionality based on psychometric networks (29). For this, partial regularized correlations were used following the graphical least absolute shrinkage and selection operator (GLASSO) estimator, allowing communities of items to be identified in the network (30). Previously, a Unique Variable Analysis (UVA) was carried out to detect potential local dependence problems among the reactive dimensions. The structural stability and that of the items was assessed with 1,000 bootstrap resamplings, following recommendations for network-based dimensionality analysis (31). Reactive items with stability indices below.70 were considered unstable and excluded before proceeding with the exploratory factor analysis.

Third, and complementary to the EGA, an exploratory factor analysis (EFA) was performed on a polychoric correlation matrix adapted for ordinal data under the assumption of underlying continuous latent variables (32). The optimal number of factors was determined through permutation-based parallel analysis (33). Promin oblique rotation was applied, assuming a correlation between latent factors (34). Sample adequacy was assessed using the Kaiser-Meyer-Olkin (KMO) index and Bartlett’s test of sphericity. Factor loadings ≥ 0.40 were considered as the minimum criterion for the retention and interpretation of items, along with the absence of substantive cross-loads and the solution’s theoretical consistency.

A confirmatory factor analysis (CFA) was subsequently carried out using the second independent subsample. Given the ordinal nature of the items and the absence of multivariate normality, the diagonally weighted least squares (DWLS) estimator, recommended for this type of data, was used (35). The model’s fit was assessed using the comparative fit index (CFI), the Tucker-Lewis index (TLI), root mean square error of approximation (RMSEA) and standardized root mean square residual (SRMR), considering CFI and TLI ≥ 0.95 and RMSEA and SRMR ≤ 0.08 to be good fit value criteria. In addition, standardized factor loadings (*λ*) were examined, considering values ≥ 0.50 to be a desirable criterion. The retained factors’ score reliability was estimated with the omega coefficient (*ω*), while convergent and discriminant validity was assessed with the average variance extracted (AVE), the Fornell-Larcker criterion and the heterotrait-monotrait (HTMT) index (36).

Lastly, factor invariance by sex was evaluated at the configurational, threshold, metric and scalar levels, considering the items’ ordinal nature (37). Invariance was considered supported when the change in CFI was ≤ 0.01 between nested models and no substantial deterioration was observed in RMSEA or SRMR (38).

All analyses were performed using R (lavaan and EGAnet packages) and FACTOR software.

## 3 Ethical considerations

This study was approved by the Universidad del Bio Bio Ethics Committee. All participants gave their written informed consent before taking part. The voluntary and anonymous nature of their participation was assured, along with the confidentiality of the information produced and its exclusive use for academic purposes. All procedures were developed in accordance with the Declaration of Helsinki for research on human beings.

## 4 Results

### 4.1 Assessment of the original model and exploration of CCRAM’S dimensionality

The first step in assessing the instrument’s internal structure involved examining the adequacy of CCRAM’s original factor model (21 items organized into five dimensions) through confirmatory factor analysis (CFA). While the solution showed acceptable global fit indices (CFI = 0.98; TLI = 0.98; RMSEA = 0.06; SRM*R* = 0.05), the inspection of local parameters showed significant limitations in the model’s psychometric quality.

In particular, average variance extracted (AVE) was below the recommended threshold of 0.50 in several dimensions — for example, in Collective Effectiveness (AVE = 0.38) and Attachment to Place (AVE = 0.44) — indicating convergent validity weaknesses. Furthermore, reliability coefficients showed heterogeneous results among factors. While some dimensions showed adequate levels of internal consistency (e.g., Collective Effectiveness, *ω* = 0.83), others registered lower values (e.g., *ω* = 0.67 in Preparedness).

In addition, high correlations were observed between some factors, with the relationship between Collective Effectiveness and Preparedness (*r* = 0.95, *p* < 0.001) standing out, suggesting insufficient empirical differentiation between these dimensions and possible problems with discriminant validity. Taken together, these results show that, despite acceptable global fit indices, the instrument’s original factor structure shows weaknesses in terms of convergent validity, discriminant validity and internal consistency.

Given these limitations, it was considered methodologically relevant to re-examine the instrument’s internal structure through a broader exploratory approach. For this an extended version of CCRAM composed of 31 reactive items was used, which includes the 28 items in CCRAM-28 (9) plus three additional items from the instrument’s initial formulation (15). This strategy allowed assessing a broader set of indicators of the community resilience construct prior to proceeding with the instrument’s factor filtering.

Where feasible, it is recommended that broader banks of items be used in the initial exploratory phases of psychometric validation studies, as it allows a broader assessment of the reactive items’ empirical behavior, identifying alternative factor configurations and systematically filtering the instrument prior to its reduction and factor confirmation (27, 39). In this framework, the inclusion of 31 items allowed the CCRAM’s dimensionality to be reassessed and a more parsimonious factor solution to be defined in this population.

To this end, the questionnaire’s internal structure was re-examined from an exploratory logic. A preliminary analysis of the items was first performed to assess their psychometric performance and identify potentially problematic indicators.

The instrument’s dimensionality was examined through two complementary strategies. First, an Exploratory Graph Analysis (EGA) was applied, a network-based technique that allows identifying communities of items based on regularized partial associations and estimating the instrument’s dimensionality (29, 31). Second, an exploratory factor analysis (EFA) was performed on a polychoric correlation matrix, a procedure recommended for ordinal items (32, 40).

### 4.2 Preliminary analysis of items

The preliminary psychometric performance of the 31 items was assessed with descriptive statistics and discrimination indices (see Table 1). Medians (*Mdn*) ranged between 3 and 4 and interquartile ranges (ICR) ranged from 1 to 2, suggesting adequate response dispersion. Likewise, asymmetry and kurtosis values remained within the ±1.5 range, without relevant univariate deviations.

**Table 1:** Descriptive statistics and item discrimination indices.

Item	Med.	IQR	Skew.	Kurt.	Corrected item-total correlation	Multiple R^2^	Ordinal alpha if deleted *α*
1	3.0	1.0	−0.4	−0.1	0.550	0.442	0.907
2	4.0	1.0	−0.6	−0.4	0.555	0.386	0.907
3	3.0	2.0	−0.2	−0.8	0.514	0.308	0.908
4	4.0	1.0	−1.3	1.7	0.444	0.390	0.909
5	4.0	1.0	−0.7	0.6	0.494	0.382	0.908
6	3.0	2.0	−0.1	−0.2	0.685	0.575	0.905
7	4.0	1.0	−0.7	0.1	0.588	0.464	0.907
8	3.0	1.0	−0.3	−0.4	0.548	0.394	0.907
9	4.0	2.0	−0.8	0.1	0.521	0.539	0.908
10	4.0	1.0	−0.4	−0.3	0.539	0.467	0.907
11	3.5	1.0	−0.5	−0.1	0.556	0.368	0.907
12	3.0	1.0	−0.4	−0.5	0.440	0.264	0.909
13	3.0	2.0	0.1	−0.8	0.390	0.264	0.910
**14**	**4.0**	**2.0**	**−1.1**	**0.3**	**0.256**	**0.179**	**0.912**
15	3.0	2.0	−0.2	−0.4	0.708	0.580	0.905
16	4.0	1.0	−0.8	0.8	0.681	0.527	0.905
17	3.0	2.0	0.1	−0.8	0.483	0.289	0.908
18	4.0	2.0	−1.0	0.0	0.423	0.422	0.909
19	3.0	1.0	0.0	−0.4	0.532	0.448	0.908
20	3.0	2.0	−0.3	−0.7	0.454	0.301	0.909
21	3.0	1.0	−0.4	−0.3	0.625	0.446	0.906
22	4.0	1.0	−0.7	−0.1	0.522	0.339	0.908
23	3.0	2.0	−0.3	−0.8	0.458	0.299	0.909
24	3.0	2.0	−0.3	−0.5	0.600	0.497	0.906
25	3.0	2.0	0.0	−1.1	0.442	0.308	0.909
**26**	**3.0**	**1.0**	**−1.1**	**0.9**	**0.061**	**0.123**	**0.915**
27	3.0	1.0	0.2	−0.9	0.374	0.273	0.910
28	3.0	2.0	−0.2	−0.6	0.581	0.466	0.907
**29**	**4.0**	**1.0**	**−1.0**	**0.9**	**0.144**	**0.159**	**0.914**
30	4.0	1.0	−0.4	0.6	0.378	0.378	0.910
**31**	**4.0**	**1.0**	**−0.6**	**−0.5**	**0.226**	**0.317**	**0.912**

Med., median; IQR, interquartile range; corrected item-total r, corrected item-total correlation; multiple R^2^, squared multiple correlation. Items 14, 26, 29, and 31, with *r* < 0.30, were excluded from subsequent dimensionality analyses. Bold values indicate items excluded from subsequent dimensionality analyses due to corrected item–total correlations below 0.30.

Item discrimination was examined with corrected item-total correlations. The majority of reactive items registered coefficients above 0.30, indicating an adequate association with the total scale score. However, the items 14 (*r* = 0.256), 26 (*r* = 0.061), 29 (*r* = 0.144) and 31 (*r* = 0.226) failed to meet this criterion, suggesting low discriminative capacity. These reactive items were consequently excluded from subsequent dimensionality analyses.

The preliminary internal consistency of the initial set of items, estimated by ordinal alpha (*α*), was high (*α* = 0.91), supporting an adequate level of internal homogeneity before the factor filtering process.

### 4.3 Exploratory graph analysis (EGA)

**Figure 1:**
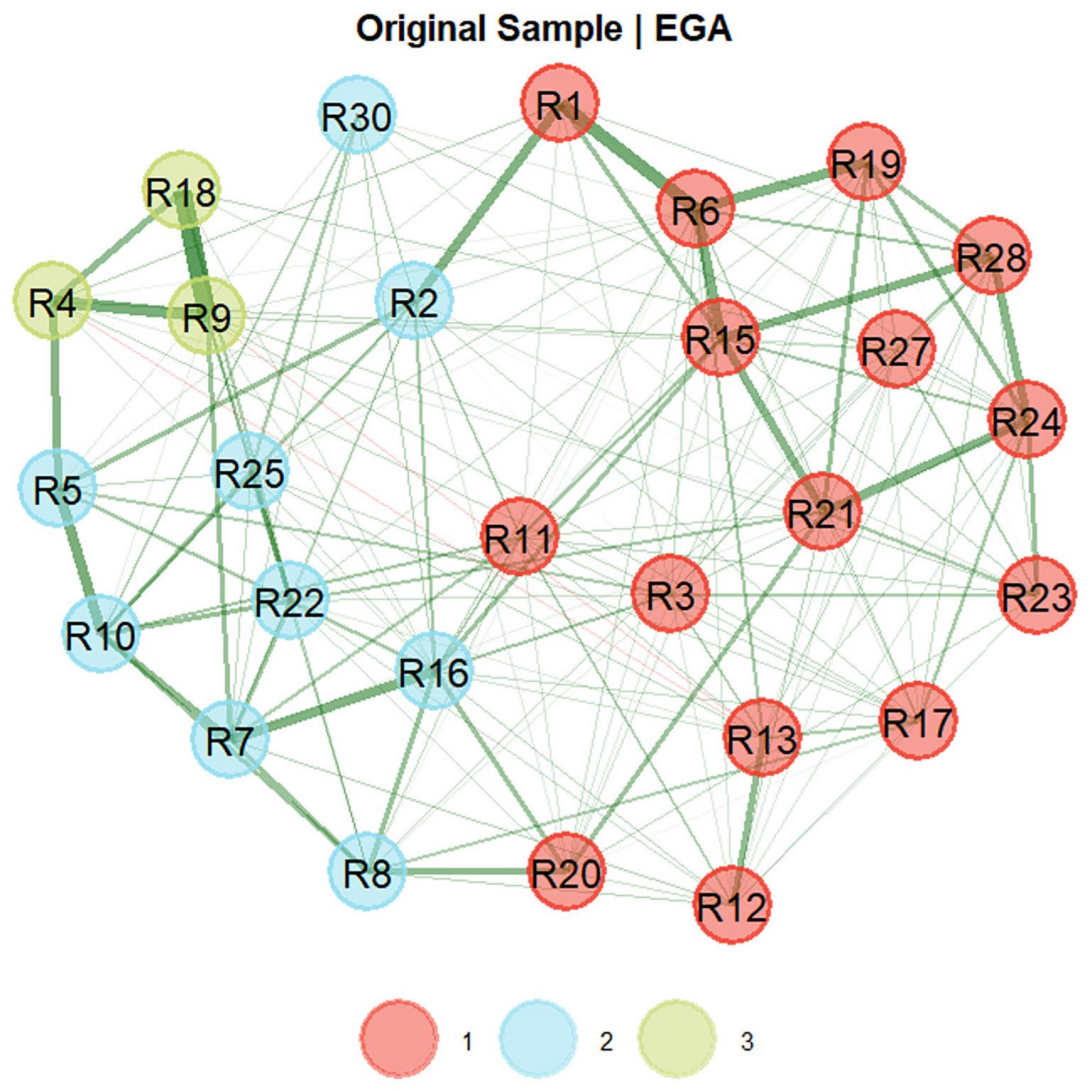
EGA-estimated community structure for the instrument’s original 27-item version.

En Exploratory Graph Analysis (EGA) was carried out on the subsample corresponding to the first survey (*n =* 384), using network estimation with the EBICglasso algorithm applied to the polychoric correlation matrix. Previously, a Unique Variable Analysis (UVA) was carried out to assess potential local dependence problems among the reactive dimensions. The results did not reveal any problematic residual associations, as partial correlations did not exceed the threshold of *r* = 0.30. The initial 27-item solution identified three communities with 173 connections and a network density of *D* = 0.493 (see Figure 1).

A bootstrap procedure with *B* = 1,000 resamplings was performed to assess dimensionality stability. The results indicate a median of three dimensions (*Mdn =* 3), with a standard error (SE) = 0.72 and a confidence interval of 95% [1.58, 4.42]. The frequency analysis showed that the three-dimensional structure was the most frequent, observed in 64% of the replicates (*p* = 0.640) when all items are considered. However, alternative solutions with four or more dimensions were also identified, suggesting a certain instability associated with some reactive items (see Table 2).

**Table 2:** Bootstrap frequency distribution of the number of dimensions estimated by EGA.

Number of dimensions	Proportion of replicates (including unstable items)	Number of dimensions	Proportion of replicates (excluding unstable items)
2	0.018	2	0.026
**3**	**0.640**	**3**	**0.916**
4	0.258	4	0.058
5	0.068		
6	0.014		
7	0.002		

Source: Authors’ compilation. Bold values indicate the retained three-dimensional solution selected based on bootstrap dimensional stability.

**Figure 2:**
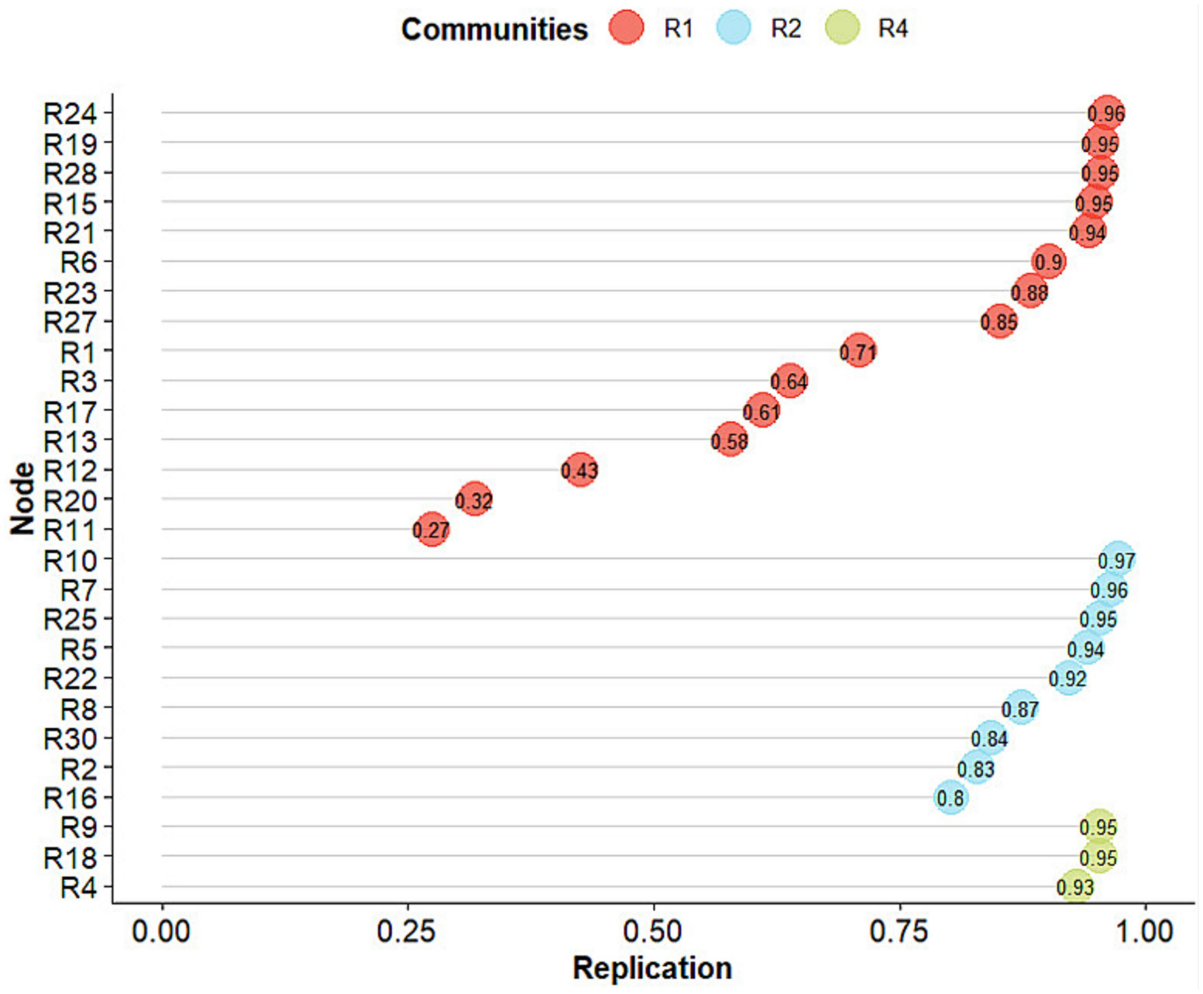
Stability of items in CCRAM’s original 27-item version.

The item stability analysis revealed that the indices of six indicators were below the recommended criterion of S < 0.*70*, specifically items 3, 11, 12, 13, 17 and 20. As can be seen in Figure 2, these reactive items showed low consistency in their allocation to communities through bootstrap resampling. These items were consequently excluded from the model, and a new network estimation was carried out.

**Figure 3:**
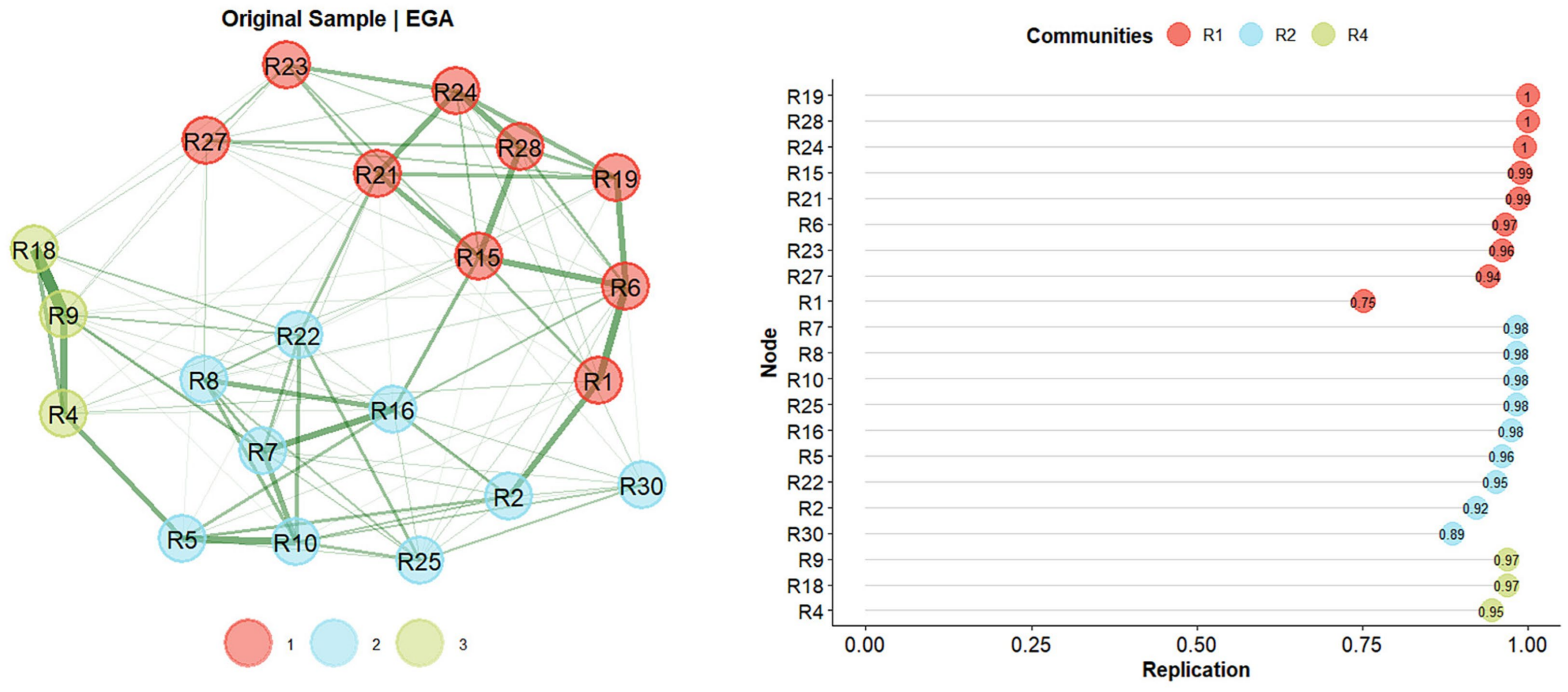
Community structure and item stability for the refined 21-item CCRAM solution.

The refined 21-item solution presented three communities with 103 connections and a network density of *D* = 0.490. The estimated community structure after filtering items is presented in Figure 3. The bootstrap resampling again confirmed a three-dimensional structure, with this solution observed in 91.6% of replicates (*p* = 0.916) (see Table 2), indicating greater dimensional stability after the removal of unstable items.

The structural consistency analysis of the communities produced values of *SC₁* = 0.948, *SC₂* = 0.716 and *SC₃* = 0.810. These values exceed the threshold of SC ≥ 0.70, considered acceptable for dimensional stability in EGA. Furthermore, the average stability of items was high (Mstaᵦ > 0.95), indicating a highly consistent allocation of the indicators to their respective communities.

Taken together, the results show that the filtering of items allowed the three-dimensional structure’s stability to increase substantially, going from 64% of the replicates in the initial 27-item solution to 91.6% in the refined 21-item solution. This increase in the frequency of the dominant dimensionality is evidence of the model’s greater structural robustness, supporting the three-dimensional structure obtained in this phase.

### 4.4 Exploratory factor analysis (EFA)

An exploratory factor analysis (EFA) was carried out on the same subsample (*n* = 384) to contrast the EGA results, using a polychoric correlation entry matrix, appropriate for ordinal items.

Sample adequacy was optimal, with a Kaiser–Meyer–Olkin index of *KMO* = 0.902. Furthermore, Bartlett’s test of sphericity was significant, χ^2^(210) = 3580.50, *p* < 0.001, confirming the factorizability of the correlation matrix. The matrix determinant was 7.16 × 10^−5^, indicating the absence of severe multicollinearity problems.

The number of factors to be retained was determined via a permutation-based parallel analysis, which indicated the retention of three dimensions (see Table 3). The three-dimensional solution explained 53.30% of total variance. Because correlation between latent dimensions was expected, oblique Promin rotation was applied. The fit indices indicated a suitable solution for the three-dimensional model (*RMSEA* = 0.031, *SRMR* = 0.043, *NNFI* = 0.992 y *CFI* = 0.994) (see Table 3).

**Table 3:** Exploratory factor analysis fit indices by number of dimensions.

Number of dimensions	RMSEA	SRMR	GFI	NNFI	CFI
1	—	0.095	0.496	—	—
2	0.059	0.065	1.000	0.970	0.976
**3**	**0.031**	**0.043**	**1.000**	**0.992**	**0.994**
4	0.000	0.033	1.000	1.002	0.999

The three-factor solution was selected based on parallel analysis and its adequate balance between parsimony and fit indices. Bold values indicate the retained three-factor solution selected based on parallel analysis and the balance between model fit and parsimony.

All retained factor loadings were ≥ 0.40 and no substantive cross-loads were observed, which supports the empirical differentiation of dimensions (see Supplementary material 2).

The three new factors’ denomination was established considering the primary factor loading pattern, the absence of relevant secondary saturations, the conceptual consistency of grouped reactive items and their articulation with the definition of the original dimension (12), in accordance with methodological recommendations for the interpretation of exploratory factor solutions (41, 42).

The first factor was denominated Territorial Attachment and Identity and was composed of items 4, 9 and 18, with factor loadings of between 0.50 and 0.84. The contents of these reactive items refer to pride in the place of residence, sense of belonging and territorial roots. Taken together, these indicators represent a dimension that is consistent with the place attachment and place identity constructs, meaning that the assigned denomination is consistent both with the items’ content as well as the factor’s empirical structure (5, 43).

The second factor was denominated Perceived Local Governance and was composed of items 1, 6, 15, 19, 21, 23, 24, 27 and 28, with factor loadings of between 0.48 and 0.81. The contents of these reactive items refer to confidence in the authorities, leadership capacity in critical situations, equity in the provision of services, operational continuity of public services in emergencies and the quality of information provided by the authorities. Taken together, these indicators represent a dimension that is consistent with perceived local governance, insofar as they express community assessments of local institutions’ performance, legitimacy and coordination capacity in critical situations (44, 45).

The third factor was denominated Community Social Capital and was composed of items 2, 5, 7, 8, 10, 16, 22, 25 and 30, with factor loadings of between 0.47 and 0.80. The contents of these reactive items refer to mutual neighborhood assistance, the quality of intergroup relations, interpersonal trust, shared knowledge about how to act in emergencies, perceived neighborhood security and belief in the collective capacity to face critical situations. Taken together, these indicators represent a dimension that is consistent with community social capital, inasmuch as they express relational resources based on trust, reciprocity and cooperation among community members (46, 47).

Overall, the factor structure obtained suggests a partial empirical reconfiguration of the dimensions proposed in the original CCRAM model. Specifically, the indicators associated with *leadership* and *preparedness* were grouped into a common dimension, interpreted as Perceived Local Governance, while indicators associated with *social trust* and *collective effectiveness* converged in a Community Social Capital dimension.

In summary, the EFA supported a three-dimensional structure with statistical adequacy and conceptual interpretability, in line with the results obtained through EGA. In this sense, the findings provide convergent evidence on the instrument’s dimensionality and an empirical basis for subjecting this structure to assessment through confirmatory factor analysis of an independent subsample.

### 4.5 Confirmatory factor analysis (CFA)

A confirmatory factor analysis (CFA) was performed on an independent subsample corresponding to the year 2024 (*n* = 545) to contrast the three-dimensional structure derived from exploratory analyses. Given the ordinal nature of the items, the model was estimated using diagonally weighted least squares (DWLS) based on a polychoric correlation matrix.

A model of three correlated factors was specified in the stage, in which each item was exclusively loaded in its theoretical dimension. The initial model showed an adequate fit, χ^2^(167) = 669.30, χ^2^/df = 4.00, CFI = 0.972, TLI = 0.968, GFI = 0.961, SRM*R* = 0.062 and RMSEA = 0.066.

A Heywood case was detected in item 30 (“My community’s level of resilience is high”) during the estimation, evidenced by a negative residual variance. This item was consequently eliminated, and the model was re-specified. The magnitude of the standardized factor loadings was subsequently examined and items 25 (*λ* = 0.32) and 27 (*λ* = 0.38) were excluded for having saturations below the 0.50 criterion. Items 23 (*λ* = 0.57) and 5 (*λ* = 0.58) were removed during an additional filtering phase, based on parsimony criteria and the relative contribution of their respective factors.

The final model was composed of 16 items distributed across three correlated factors (Table 4). All standardized factor loadings were statistically significant (*p* < 0.001). Loads ranged between 0.62 and 0.86 in Factor 1, Territorial Attachment and Identity; between 0.66 and 0.77 in Factor 2, Perceived Local Governance; and between 0.61 and 0.75 in Factor 3, Community Social Capital. The descriptive statistics of the items showed medians of between 3 and 4 and interquartile ranges between 1 and 2. The final Spanish version of the scale is provided as Supplementary material 3.

**Table 4:** Descriptive statistics and standardized factor loadings for the final 16-item model.

Factor	Item	Me	IQR	λ	SE	*p*	95% CI
F1. Place attachment and territorial identity	4. I feel proud to say where I am from	4	1	0.75	0.02	< 0.001	[0.72–0.79]
	9. I feel a strong sense of belonging to my place of residence	4	1	0.86	0.02	< 0.001	[0.82–0.89]
	18. I would regret leaving the place where I live	4	2	0.62	0.02	< 0.001	[0.59–0.65]
F2. Perceived local governance	1. The regional authority where I live functions well	3	2	0.68	0.01	< 0.001	[0.66–0.71]
	6. I fully trust those responsible for regional authority	3	2	0.77	0.01	< 0.001	[0.75–0.79]
	15. I trust the ability of the regional authority to lead during crises	3	2	0.76	0.01	< 0.001	[0.74–0.78]
	19. Regional authorities provide services fairly	3	1	0.70	0.02	< 0.001	[0.66–0.74]
	21. I trust the continuity of regional services during emergencies	3	1	0.75	0.02	< 0.001	[0.73–0.77]
	24. The information provided by authorities during emergencies meets my needs	3	2	0.72	0.02	< 0.001	[0.70–0.74]
	28. Municipal officials demonstrate leadership	3	2	0.66	0.01	< 0.001	[0.64–0.69]
F3. Community social capital	2. There is mutual help and concern where I live	3	1	0.64	0.02	< 0.001	[0.61–0.67]
	7. I trust that people in my community will help me in a crisis	4	1	0.63	0.02	< 0.001	[0.60–0.65]
	8. Neighbors know what to do in an emergency	3	2	0.61	0.02	< 0.001	[0.58–0.63]
	10. In my neighborhood, neighbors trust one another	3	2	0.61	0.02	< 0.001	[0.58–0.63]
	16. I trust my community’s ability to overcome emergencies	4	1	0.75	0.02	< 0.001	[0.73–0.78]
	22. I feel safe in my community	4	1	0.65	0.02	< 0.001	[0.62–0.68]

Me, median; IQR, interquartile range; λ, standardized factor loading; SE, standard error; CI, confidence interval. Items were originally administered in Spanish. English wording is provided for reporting purposes. Source. Authors’ compilation.

In terms of global fit, the final model presented adequate indices, CFI = 0.980, TLI = 0.976, GFI = 0.986, SRM*R* = 0.060 and RMSEA = 0.069, supporting the three-dimensional solution’s empirical adequacy. Furthermore, interfactor correlations were moderate (r_12_ = 0.42, r_13_ = 0.63 and r_23_ = 0.71), showing a relationship between dimensions without excessive redundancy.

Regarding internal consistency, compound reliability (*ω*) was 0.77 for Factor 1, 0.87 for factor 2 and 0.80 for Factor 3. For its part, average variance extracted (AVE) was 0.56, 0.52 and 0.42, respectively. Though the third factor registered an AVE below the conventional cutoff point of 0.50, its retention was considered admissible given its level of compound reliability and the observed pattern of interfactor associations.

Discriminant validity was assessed using the Fornell-Larcker criterion and the HTMT index (Table 5). The square root of the average extracted variance (√AVE) was higher than the interfactor correlations in Factors 1 and 2. However, this criterion was not strictly met in Factor 3 with respect to Factor 2, given that the observed interfactor correlation (0.71) exceeded the factor’s √AVE (0.65). However, HTMT values remained below the recommended threshold of 0.85, providing additional evidence in favor of the proposed structure’s discriminant validity.

**Table 5:** Interfactor correlations, composite reliability, average variance extracted, and discriminant validity evidence.

Factor	F1	F2	F3	ω	AVE
F1. Place attachment and territorial identity	**0.75**			0.77	0.56
F2. Perceived local governance	0.42***	0.**72**		0.87	0.52
F3. Community social capital	0.63***	0.71***	**0.65**	0.80	0.42

Bold diagonal values correspond to the square root of the average variance extracted (√AVE). Interfactor correlations are shown below the diagonal. ***p < 0.001.

Taken together, these findings support confirmatory evidence favoring the instrument’s three-dimensional structure in an independent sample.

### 4.6 Analysis of factor invariance by sex

A multigroup factor invariance analysis was carried out using DWLS on a polychoric correlation matrix to assess the equivalence of the 16-item three-factor model between men and women. Configurational, threshold, metric and scalar invariance were examined sequentially (see Table 6).

**Table 6:** Factor invariance analysis.

Invariance	χ^2^	df	∆χ^2^	∆df	*p*	CFI	∆CFI	SRMR	RMSEA
Configural	470.6	202				0.980		0.068	0.070
Thresholds	487.8	234	17.2	32	0.503	0.981	0.001	0.068	0.063
Metric	503.2	247	15.4	13	0.692	0.981	0.000	0.068	0.062
Scalar	503.2	247	0	0	1.00	0.981	0.000	0.068	0.062

Source. Authors’ compilation.

The configurational model showed an adequate fit, χ^2^(202) = 470.60, CFI = 0.980, SRM*R* = 0.068 and RMSEA = 0.070, which supports replication of the factor structure in both groups.

Threshold invariance was also supported, as the restricted model showed no significant deterioration compared to the configurational one, Δχ^2^ (32) = 17.20, *p* = 0.503, ΔCFI = 0.001, with adequate fit indices (CFI = 0.981, SRM*R* = 0.068, RMSEA = 0.063).

Metric invariance was subsequently supported by the absence of relevant changes in the fit after imposing equality in factor loadings, Δχ^2^ (13) = 15.40, *p* = 0.692 and ΔCFI = 0.000, maintaining adequate global fit values (CFI = 0.981, SRM*R* = 0.068, RMSEA = 0.062).

Lastly, the scalar model did not show differences with the metric one, Δχ^2^(0) = 0, *p* = 1.00 and ΔCFI = 0.000, retaining the same fit indices (CFI = 0.981, SRM*R* = 0.068, RMSEA = 0.062).

Taken together, the results support the model’s configurational, threshold, metric and scalar invariance, which supports the comparability of latent scores between men and women.

## 5 Discussion

This study examined CCRAM’s psychometric properties in the Chilean population and provided favorable evidence for the use of an abbreviated 16-item version structured around three correlated factors (the final scale is presented in Supplementary material 3). In general terms, the results support validity evidence based on internal structure, adequate score reliability, discriminant validity between factors and invariance measured by sex. However, the findings do not support replication of the original five-factor structure in this population. This finding is consistent with a contemporary conception of validity, understood not as a fixed attribute of the instrument but as an argument based on empirical and theoretical evidence that supports the interpretation of scores for specific uses and contexts (13).

While the model’s initial assessment showed acceptable global fit indices, it also revealed relevant psychometric weaknesses at the local level, including low average variance extracted in some dimensions, heterogeneous reliability and an excessively high correlation between collective effectiveness and preparedness. Consequently, the global fit was insufficient to support the original model’s fit in the Chilean context due to persistent problems of convergent validity, interfactor differentiation and internal consistency in part of its dimensions. It was therefore necessary to reexamine the instrument’s dimensionality through the exploratory and confirmatory analysis of independent subsamples. In line with the Standards for Educational and Psychological Testing, this result underscores the need to assess the internal structure and the consistency between the construct’s empirical operationalization and its theoretical definition before supporting a measure’s validity in a new population (13, 14).

These differences can, in part, be interpreted in light of the limitations of generalizing measurement structures originally developed in WEIRD contexts. A significant portion of psychological research has been built on western, educated, industrialized, rich and democratic samples, limiting the automatic extrapolation of factor configurations to different cultural, territorial and institutional contexts (21). However, the fact that the original CCRAM structure cannot be reproduced in the Chilean population should not be interpreted as a failure on the part of the instrument, but instead as an expected result in a process of cross-cultural adaptation to examine the construct’s conceptual equivalence, the contextual relevance of its indicators and the stability of its internal structure in a new population (13, 20, 21). This consideration is particularly relevant in a context like the Chilean one, characterized by institutional centralization in DRM, territorial inequality and recurrent exposure to socionatural threats, where the resources associated with community resilience can be empirically organized in a way that differs from the context in which the instrument was originally developed.

The convergence of EGA and EFA in a single three-factor solution reinforces the consistency of the structure identified and supports the relevance of the retained solution. In this framework, the filtering of reactive items contributed to delimiting a more parsimonious version of the instrument, preserving the indicators with greater consistency within the observed empirical organization (23, 27, 39).

At the dimensional level, the regrouping of indicators originally associated with leadership and preparedness into a single factor suggests that preparedness is not configured as a differentiated domain from local risk governance in this population. Rather, the two components appear to become integrated in a broader appreciation of institutional performance in leading, coordinating and sustaining DRM. From this perspective, the new dimension of perceived local governance expresses a community assessment of how authorities and institutions articulate actors, communicate risk, ensure operational continuity and deploy capacities to address critical scenarios (44, 45). The incorporation of item 28 is also relevant. Originally considered a complementary contextual indicator in CCRAM-28, in this sample it was integrated into this factor because of its reference to municipal leadership (9). This inclusion suggests that the appreciation of institutional performance is not restricted to formal preparedness components but that it also includes situated appreciation of concrete territorial leaderships. Taken together, this pattern is consistent with the literature situating these capacities as core components of community resilience and DRM (9, 44, 45). In the Chilean context, this suggests that preparedness tends to be less associated with an attribute limited to the individual or family levels and more an expression of territorial risk governance capacity.

Furthermore, the integration in a single factor of indicators associated with the social trust and collective effectiveness dimensions of CCRAM-28, along with contents related to mutual support and shared knowledge in the face of emergencies, suggests that these resources do not emerge as empirically differentiated domains in this population. Rather, they appear to be articulated as interdependent expressions of a more agential and relational dimension of community resilience (48). From this perspective, community social capital can be understood as a latent configuration based on interpersonal trust, reciprocity, everyday cooperation and shared expectations of joint action, resources widely recognized as socio-community foundations for coping with adverse events (3, 44–46). Unlike the previous dimension, focused on institutional capacities and risk governance, this factor organization emphasizes the response capacity that emerges from the community fabric itself. That is, from bonds of proximity, support networks and shared perceptions of collective effectiveness. This interpretation is consistent with the literature showing the close articulation between trust, mutual support and collective effectiveness in risk and disaster contexts (44, 46, 47).

For its part, the permanence of a specific territorial attachment and identity factor indicates that the connection to place retains an empirically differentiated configuration within the instrument’s structure. Unlike the structural-institutional dimension described above and the agential-relational dimension associated with community social capital, this factor refers to a dimension of a territorial, symbolic and affective-relational nature. Its content groups together indicators related to the sense of belonging, pride in the place of residence and territorial rootedness, meaning that it maintains continuity with CCRAM-28’s original dimension of attachment to place, though its scope is expanded in this sample by also incorporating territorial identification processes. This reorganization is consistent with the literature on place attachment and place identity, which has shown that affective and symbolic connections to the territory constitute relevant resources to sustain permanence, organization and continuity of community life in adverse contexts (5, 43).

Overall, the confirmatory phase supported the final 16-item solution with three correlated factors identified in the foregoing analysis, contributing additional evidence of the structure’s consistency in an independent subsample. The progressive filtering of reactive items allowed a more parsimonious model to be consolidated without modifying the instrument’s substantive organization. While one of the factors registered an average extracted variance below the conventional cut-off point, its retention is justified by the acceptable magnitude of compound reliability, the significance of factor loadings and the dimension’s conceptual consistency within the model (35, 36, 49). Likewise, the discriminant validity and invariance by sex analyses support both the empirical differentiation between factors as well as the measurement structure’s equivalence between men and women, strengthening score interpretation in this population (20, 37, 38). In the same vein, discriminant validity was supported, which indicates that factors maintain sufficient empirical differentiation, even though they share variance as expressions of a common construct. For its part, configurational, threshold, metric and scalar invariance by sex support the model’s equivalence among men and women and allows comparisons of latent scores between the two groups (20, 37, 38).

## 6 Practical interpretation and intended uses of the Chilean CCRAM scores

From the perspective of the *Standards for Educational and Psychological Testing*, validity refers to the degree to which available evidence supports the use and interpretation of scores for specific purposes, rather than to an inherent property of the instrument itself (10). Accordingly, the findings of this study support the use of the Chilean version of CCRAM as an indicator of perceived community resilience among Chilean adults, operationalized through three dimensions: Territorial Attachment and Identity, Perceived Local Governance, and Community Social Capital.

In previous studies, CCRAM has been used to profile perceived community resilience, compare communities or territories, monitor changes over time, and identify strengths and weaknesses in specific dimensions related to leadership, collective efficacy, preparedness, place attachment, and social trust (6, 12). Consistent with these uses, the Chilean version may contribute to research, community diagnosis, territorial monitoring, and the evaluation of interventions aimed at strengthening capacities associated with disaster risk reduction. In practical terms, relatively lower scores in specific dimensions may help identify areas requiring targeted action, such as strengthening local governance, community social capital, or place-based attachment and belonging. Periodic application of the instrument may also contribute to monitoring changes in perceived community resilience and evaluating community-strengthening initiatives in contexts exposed to risks and disasters.

However, the use and interpretation of scores should remain limited to the evidence gathered in this study. The instrument does not provide an absolute measure of resilience, nor does it allow communities to be classified as inherently resilient or non-resilient. Rather, scores should be understood as contextually situated indicators of perceived community resilience, whose interpretation may vary according to the type of hazard, territorial conditions, and institutional arrangements in which communities are embedded. Because this study did not establish normative cut-off scores or criterion-based classifications, comparisons should be interpreted as relative differences in perceived community resilience rather than as diagnostic thresholds. Further evidence regarding relationships with external variables, temporal stability, and applicability in other territorial and risk contexts is needed to expand the range of empirically supported uses and interpretations. Therefore, the Chilean CCRAM should be understood as a tool for supporting evidence-based decisions and interpretations in disaster risk management research and practice, rather than as a definitive or context-free index of community resilience.

## 7 Conclusion

To conclude, CCRAM’s cross-cultural adaptation to the Chilean population did not support replication of the original factor structure but allowed identifying an abbreviated 16-item version with favorable validity evidence based on the internal structure, adequate reliability and invariance by sex. The resulting solution, organized into three correlated factors (Territorial Attachment and Identity, Perceived Local Governance and Community Social Capital), suggests an empirical reorganization of the construct in this application context. In this sense, the version obtained constitutes a brief, parsimonious and contextually relevant measure for assessing community resilience in the Chilean adult population. However, the interpretation of its scores should be limited to the evidence compiled in this study. For this reason, future research should expand its validation through the analysis of temporal stability, the relationship with external variables and evaluation in other territorial contexts and types of risk.

## Data Availability

The datasets generated and analyzed during the current study are not publicly available because they are being used in ongoing research projects and additional manuscripts derived from the same research program. However, the Supplementary Material accompanying this article includes the linguistic and conceptual adaptation details, supplementary psychometric analyses, and the final Spanish version of the scale. Requests for additional information may be directed to the corresponding author.
