# Supplementary material for: Cross-cultural adaptation and validation of the Conjoint Community Resiliency Assessment Measure (CCRAM) among Chilean adults
Source: Front Public Health. 2026 Jun 26;14:1866030. doi: 10.3389/fpubh.2026.1866030 (PMC13350333; doi:10.3389/fpubh.2026.1866030)
Supplement: Supplementary file 1 [file Table_1.DOCX]

**Supplementary Table S1**
*Linguistic and Conceptual Adaptations Made to the Spanish Version of the CCRAM for Use in Chilean Participants*

| **Type of adaptation / Tipo de adecuación** | **Description / Descripción** | **Examples of modified items / Ejemplos de reactivos modificados** | **Purpose / Propósito** |
| --- | --- | --- | --- |
| Lexical and syntactic simplification / Simplificación léxica y sintáctica | Less natural or overly formal expressions were replaced with wording more commonly used in local Spanish. / Se reemplazaron expresiones menos naturales o más formales por formulaciones de uso más habitual en el español local. | “mi lugar de residencia” → “donde vivo” (Items 1, 2); “resido” → “vivo” (Ítem 18); “residentes” → “vecinos” (Ítems 8, 10, 20) | To improve item comprehensibility and fluency. / Mejorar comprensibilidad y fluidez de los reactivos. |
| Adaptation of territorial references / Adecuación de referentes territoriales | General expressions were replaced with terms more contextually appropriate for Chilean use. / Se sustituyeron expresiones generales por términos más próximos al uso contextual chileno. | “lugar de residencia” → “localidad”, “vecindario”, “comunidad” (Ítems 3, 10, 11, 22, 23, 26) | To increase semantic and contextual relevance. / Aumentar pertinencia semántica y contextual. |
| Adaptation of institutional references / Adecuación de referentes institucionales | References to authorities and services were adjusted to improve contextual appropriateness. / Se ajustaron referencias a autoridades y servicios para mejorar su adecuación al contexto de aplicación. | “autoridad municipal” → “autoridad regional” (Ítems 1, 19, 24); “alcalde” → “autoridad regional” (Ítem 15) | To improve contextual equivalence of items. / Favorecer equivalencia contextual de los reactivos. |
| Semantic reformulation / Reformulación semántica | Some items were strengthened or specified to improve clarity. / Se reforzó o precisó el contenido de algunos reactivos para mejorar su claridad. | “Siento pertenencia…” → “Siento un fuerte sentido de pertenencia…” (Ítem 9); “personas que pueden ayudar” → “personas capacitadas” (Ítem 12) | To increase semantic precision. / Aumentar precisión semántica. |
| Conceptual reformulation / Reformulación conceptual | The focus of some items was modified to improve conceptual relevance in the Chilean context. / Se modificó el enfoque de algunos reactivos para mejorar su pertinencia conceptual en el contexto chileno. | “razones ideológicas” → “razones políticas o religiosas” (Ítem 14); “intentaré dejar…” → “consideraría la posibilidad de evacuar…” (Ítem 26); future service functioning → emergency preparedness (Ítem 23) | To improve conceptual relevance and reduce ambiguity. / Mejorar pertinencia conceptual y reducir ambigüedad. |
| Style and neutrality adjustments / Ajustes de estilo y neutralidad | Minor changes were introduced in style, inclusiveness, and syntax. / Se introdujeron cambios menores de estilo, inclusividad y orden sintáctico. | “Me siento seguro” → “Me siento seguro/a” (Ítem 22); reordenamiento sintáctico (Ítem 5) | To improve clarity and acceptability. / Mejorar claridad y aceptabilidad. |

*Note.* Adaptations were made to the Spanish version of the CCRAM used in this study in order to improve its semantic and contextual relevance for Chilean participants. Before field administration, the adapted version was pilot-tested in 30 university students to assess item comprehensibility and contextual adequacy.
