# Supplementary material for: Cross-cultural adaptation and validation of the Conjoint Community Resiliency Assessment Measure (CCRAM) among Chilean adults
Source: Front Public Health. 2026 Jun 26;14:1866030. doi: 10.3389/fpubh.2026.1866030 (PMC13350333; doi:10.3389/fpubh.2026.1866030)
Supplement: Supplementary file 2 [file Table_2.DOCX]

**Supplementary Table S2**
Exploratory Factor Loading Matrix of the 21-Item CCRAM Solution After Promin Rotation

| **Item (Spanish / English)** | **F1** | **F2** | **F3** | **h²** |
| --- | --- | --- | --- | --- |
| **Place Attachment and Territorial Identity / Apego e Identidad Territorial** |  |  |  |  |
| 18. Lamentaría dejar el lugar donde vivo / *I would regret leaving the place where I live* | .84 |  |  | .71 |
| 9. Siento un fuerte sentido de pertenencia a mi lugar de residencia / *I feel a strong sense of belonging to my place of residence* | .81 |  |  | .66 |
| 4. Me siento orgulloso/a de decir de dónde soy / *I feel proud to say where I am from* | .50 |  |  | .25 |
| **Perceived Local Governance / Gobernanza Local Percibida** |  |  |  |  |
| 24. La información proporcionada por autoridades durante emergencias cumple mis necesidades / *The information provided by authorities during emergencies meets my needs* |  | .81 |  | .66 |
| 28. Los funcionarios de la municipalidad demuestran liderazgo / *Municipal officials demonstrate leadership* |  | .80 |  | .64 |
| 15. Confío en la habilidad de la autoridad regional para liderar en tiempos de crisis / *I trust the ability of the regional authority to lead in times of crisis* |  | .77 |  | .59 |
| 19. Las autoridades regionales ofrecen servicios de manera justa y equitativa / *Regional authorities provide services in a fair and equitable manner* |  | .73 |  | .53 |
| 6. Confío plenamente en los responsables de la autoridad regional / *I fully trust those responsible for the regional authority* |  | .72 |  | .52 |
| 1. Donde vivo la autoridad regional funciona bien / *Where I live, the regional authority functions well* |  | .62 |  | .38 |
| 21. Confío en la continuidad de servicios regionales en emergencias / *I trust the continuity of regional services during emergencies* |  | .62 |  | .38 |
| 23. Los servicios de salud locales están preparados para emergencias / *Local health services are prepared for emergencies* |  | .50 |  | .25 |
| 27. El transporte público funcionará en caso de emergencia / *Public transportation will function in the event of an emergency* |  | .48 |  | .23 |
| **Community Social Capital / Capital Social Comunitario** |  |  |  |  |
| 10. En mi vecindario los vecinos confían unos en otros / *In my neighborhood, neighbors trust one another* |  |  | .80 | .64 |
| 7. Confío en que las personas de mi comunidad me ayudarán en crisis / *I trust that people in my community will help me in a crisis* |  |  | .68 | .46 |
| 16. Confío en la capacidad de mi comunidad para superar emergencias / *I trust my community’s ability to overcome emergencies* |  |  | .62 | .38 |
| 2. Donde vivo hay ayuda y preocupación mutua / *Where I live, there is mutual help and concern* |  |  | .61 | .37 |
| 5. Hay buenas relaciones entre grupos en mi comunidad / *There are good relationships among groups in my community* |  |  | .57 | .32 |
| 25. Muchos de mis vecinos son mis amigos / *Many of my neighbors are my friends* |  |  | .56 | .31 |
| 8. Los vecinos saben qué hacer en una emergencia / *Neighbors know what to do in an emergency* |  |  | .52 | .27 |
| 22. Me siento seguro/a en mi comunidad / *I feel safe in my community* |  |  | .51 | .26 |
| 30. El nivel de resiliencia de mi comunidad es alto / *The level of resilience in my community is high* |  |  | .47 | .22 |
| **Explained variance (%)** | **36.1** | **9.8** | **7.4** |  |

**Note.** Factor loadings ≥ .40 after Promin rotation are shown. F1 = Place Attachment and Territorial Identity; F2 = Perceived Local Governance; F3 = Community Social Capital; h² = Communality.
