# Supplementary material for: Cross-cultural adaptation and validation of the Conjoint Community Resiliency Assessment Measure (CCRAM) among Chilean adults
Source: Front Public Health. 2026 Jun 26;14:1866030. doi: 10.3389/fpubh.2026.1866030 (PMC13350333; doi:10.3389/fpubh.2026.1866030)
Supplement: Supplementary file 3 [file Table_3.DOCX]

**Conjoint Community Resiliency Assessment Measure (CCRAM) en población chilena en formato aplicable**

Esta sección del cuestionario se centra en recoger su opinión sobre el área donde principalmente vive. Lea cada enunciado atentamente y seleccione solo una respuesta que represente con mayor precisión su grado de acuerdo o desacuerdo.

|  | **¿En qué medida está de acuerdo con las siguientes frases?** | Totalmente en desacuerdo | En desacuerdo | Ni acuerdo ni desacuerdo | De  acuerdo | Totalmente de acuerdo |
| --- | --- | --- | --- | --- | --- | --- |
| 1 | Donde vivo la autoridad regional funciona bien. | 1 | 2 | 3 | 4 | 5 |
| 2 | Donde vivo hay ayuda y preocupación mutua. | 1 | 2 | 3 | 4 | 5 |
| 3 | Me siento orgulloso/a de decir de dónde soy. | 1 | 2 | 3 | 4 | 5 |
| 4 | Confío plenamente en los responsables de la autoridad regional de mi área. | 1 | 2 | 3 | 4 | 5 |
| 5 | Confío en que las personas de mi comunidad me ayudarán en caso de crisis. | 1 | 2 | 3 | 4 | 5 |
| 6 | Los vecinos saben qué hacer en una situación de emergencia. | 1 | 2 | 3 | 4 | 5 |
| 7 | Siento un fuerte sentido de pertenencia a mi lugar de residencia. | 1 | 2 | 3 | 4 | 5 |
| 8 | En mi vecindario, los vecinos confían unos en otros | 1 | 2 | 3 | 4 | 5 |
| 9 | Confío en la habilidad de la autoridad regional para liderar en tiempos de crisis. | 1 | 2 | 3 | 4 | 5 |
| 10 | Confío en la capacidad de mi comunidad para superar situaciones de emergencia. | 1 | 2 | 3 | 4 | 5 |
| 11 | Lamentaría dejar el lugar donde vivo. | 1 | 2 | 3 | 4 | 5 |
| 12 | Las autoridades regionales ofrecen sus servicios de manera justa y equitativa. | 1 | 2 | 3 | 4 | 5 |
| 13 | Confío en que seguirán los servicios regionales incluso durante situaciones de emergencia en mi vecindario. | 1 | 2 | 3 | 4 | 5 |
| 14 | Me siento seguro/a en mi comunidad. | 1 | 2 | 3 | 4 | 5 |
| 15 | La información proporcionada por las autoridades regionales durante emergencias cumple con mis necesidades. | 1 | 2 | 3 | 4 | 5 |
| 16 | Los funcionarios de la municipalidad donde vivo demuestran liderazgo. | 1 | 2 | 3 | 4 | 5 |
